# Partial Substitution of Maize for Sorghum With or Without Supplemental Hydrolysable Tannins on Digestibility and Postprandial Glycemia in Adult Dogs

**DOI:** 10.3389/fvets.2021.667411

**Published:** 2021-05-21

**Authors:** Liege Teixeira, Caroline Fredrich Dourado Pinto, Geruza Silveira Machado, Alexandre de Mello Kessler, Luciano Trevizan

**Affiliations:** Laboratório de Ensino Zootécnico, Department of Animal Science, Universidade Federal Do Rio Grande Do Sul, Porto Alegre, Brazil

**Keywords:** carbohydrate, tannins, phenolic compounds, digestibility, postprandial response

## Abstract

The effect of partial substitution of maize for sorghum, containing condensed tannins (CT), with or without the addition of a purified hydrolysable tannin extract (HT), on dog apparent digestibility and glycemic response were evaluated. The trial was conducted with eight adult dogs distributed in four treatments: (M) 50% maize; (MS) 25% maize + 25% sorghum; (MHT) 50% maize + 0.10% HT; (MSHT) 25% maize + 25% sorghum + 0.10% HT; in a balanced incomplete Latin square design in three periods, with two dogs per diet, per period. Data were analyzed by ANOVA procedure and glycemic response by repeated measures ANOVA over time (*P* < 0.05). The phenolic compounds analyzed were not detected after extrusion process, with a reduction mainly in diets containing sorghum. There were no differences in the digestibility coefficients of nutrients and energy between the dietary treatments (*P* > 0.05). Fecal and urinary characteristics were not changed by the addition of sorghum and HT (*P* > 0.05). The fecal score remained within the ideal classification as hard, dry, firm stools. A moderate increase in fecal pH was observed on dogs fed diets containing sorghum (*P* = 0.0948). Additionally, the partial replacement of maize for sorghum associated or not with HT do not alter the glycemic aspects evaluated among dietary treatments (*P* > 0.05). Availability of nutrients from maize and sorghum were similar. Tannins did not interfere in the nutritional capacity of the ingredients.

## Introduction

Cereal grains are widely used in pet food as sources of energy due to its starch content. In addition, starch is fundamental for appropriate extrusion process and kibble characteristics, such as expansion, cellular structure formation, and crispness (1). The most common carbohydrate sources used for companion animal products are rice, maize, and sorghum due to its wide distribution, easy acquisition, and low cost. Besides, all these ingredients are well-accepted, digested, and metabolized for dogs.

However, the evaluation of functional ingredients with appropriate nutritional characteristics that combines some beneficial effect, such as modulation of postprandial glycemic response, has been the major goal in pet food in the past years. The increasing prevalence of diseases such as obesity and diabetes are the main reasons to find other ingredients with functional properties for companion animals (2, 3). This highlights the importance of selecting ingredients based on their nutritional potential and their effects on digestion and metabolism.

In this way, sorghum becomes an interesting alternative to rice and maize because the grain contains functional properties such as groups of phenolic compounds. The most part of them are condensed tannins, secondary compounds of plant metabolism that may be active to improve health. There are some studies relating tannins as an antimicrobial, antiparasitic, antioxidant, anti-inflammatory, and antiviral agent (4). Tannins are classified as condensate (CT) or hydrolysable (HT). CTs, also known as proanthocyanidins, are polymers of flavan-3-ols and flavan-3,4-diols and, after oxidation, yields anthocyanidins (5), while HT are composed of simple phenols, gallotannins, and elagitannins, which, after hydrolysis, yields gallic and ellagic acid (6). Sorghum contains only CT (7). HT can be found in leaves, flowers, twigs, bark of some plants, and as a purified commercial extract. Additionally, sorghum varieties are divided according to their genetics and chemical composition. Sorghum type I have low level of phenols and tannins (0.28 g/kg of tannins), while types II and III present 4.48 and 11.95 g/kg of tannins, respectively (8).

In opposite to its beneficial effects, tannins can form complexes with substances present in saliva promoting astringency and food refusal (9). In addition, tannins can inhibit enzymes and form complexes with carbohydrates, proteins, and metal ions, thus impairing digestibility (10). Indeed, some studies had showed controversial results; some did not find differences between dogs fed maize- or sorghum-based diets (11, 12), while others found some differences on digestibility of dry matter (DM), organic matter, and crude protein (CP) (13), or even an increase on digestibility of CP, gross energy, and DM compared to maize diet (14). Regarding fecal characteristics, studies demonstrated that there are no differences between diets based on maize and sorghum. Finally, Carciofi et al. (11) demonstrated that sorghum-based diet promoted later postprandial meal response in dogs, thus reducing glycemic peak and favoring glycemic control. Concerning HT, Teixeira et al. (15) observed an increase on the fecal dry matter of dogs fed a diet based on rice and HT and a reduction in metabolizable energy on dogs fed a rice, sorghum, and HT diet.

We went through the evaluation of sorghum and rice blends and its effects on digestibility and glycemic index in dogs. The replacement of rice with sorghum reduced the digestibility of protein and also reduced the metabolizable energy content of the diets (15). As a partial replacement of rice, no variation in the glycemic response was observed.

Based on the fact that maize does not contain significant amounts of CT than sorghum does, we hypothesized that by mixing both, we could improve the content of tannins in the final diet, and then, it can have an impact on the glycemic index of the final diet. In addition, the impact of HT on maize must be tested to evaluate if it changes either digestibility or glycemic index. Therefore, this study evaluated the effects of the partial substitution of maize with sorghum containing CT plus the addition of a HT extract on the digestibility of nutrients and energy, fecal, and urinary characteristics, and lastly the glycemic response in adult dogs.

## Materials and Methods

All animal care and handling procedures were approved by The Institutional Animal Care and Use Committee at the Universidade Federal do Rio Grande do Sul, protocol number 26275.

### Animals

Eight healthy adult Beagle (four males and four females), coming from the Animal Science Department, Universidade Federal do Rio Grande do Sul, Porto Alegre, Brazil, were used in this study. They were all intact, between 2 and 3 years old, weighing 12.4 ± 0.97 kg, with a body condition score (BCS) ranging from 4.5 to 5.5 out of 9 points (16), made by a single trained person, and free of endo- and ectoparasites. All dogs were regularly immunized and submitted to clinical and laboratory tests to measure complete blood count (CBC) and to perform biochemical and coproparasitological analyses before the start of the study. The dogs were housed in individual stainless steel metabolic cages (1.0 × 1.0 × 1.5 m) equipped with a feces and urine collector, feeders, and drinkers, in a controlled room at 24°C, with a light/dark cycle of 14:10 h. The adaptation and positive reinforcement were used to avoid stress during the assay. 6 months before starting the trial, dogs were adapted to the metabolic cages and blood collections. During this period, they were fed twice daily inside the metabolic cages and stayed there all through the night. During the day, dogs remained in a patio playing all together for socialization. In the morning and afternoon, before being fed, dogs were set over a table for 3 min. Dogs were safely held, and a blood collection was simulated with no needle introduction; then, dogs were pet and received food. Between each trial, dogs were rested for 15 days, maintained in the patio, and playing together. At the end of the trials, dogs were castrated and given for adoption.

### Diets

Maize was partially substituted with sorghum as a way of introducing CT into the diets. Additionally, purified HT obtained from a commercial extract of the chestnut bark (*Castanea sativa*) was included into the diets. The extract was a water-soluble fine brown powder containing HT, hydrolysable polyphenols, cellulose, hemicellulose, simple sugars, lignin, minerals, and 8% moisture; its fiber content was <3%, it had a relative density of 0.5–0.6 g/mL and pH < 4.0. Four experimental diets were formulated and extruded to be isonutritives: (M) 50% maize; (MS) 25% maize + 25% sorghum; (MHT) 50% maize + 0.10% HT; (MSHT) 25% maize + 25% sorghum + 0.10% HT (Tables 1, 2). The dogs were fed twice a day (at 0830 and 1,700 h) to meet the energetic and nutritional requirements of adult dogs, as recommended by the NRC (17). Food intake was adjusted according to the body weight, weekly, in order to maintain the BCS in 5 points out of 9. The leftovers were collected, weighed, and discounted to calculate consumption. Water was provided *ad libitum*.

**Table 1:** Chemical composition of sorghum (*Sorghum bicolor* L. Moench).

**Item, % DM basis**	**Sorghum**
Dry matter	86.9
Starch	63.6
Crude protein	7.59
Total dietary fiber	15.3
Fat	2.56
Ash	1.42
Crude fiber	0.72
Gross energy, kcal/kg	4.446
Polyphenol tannins, %	4.8
Polyphenol non-tannins, %	2.6

*DM, dry matter*.

**Table 2:** Ingredients and chemical composition of experimental diets.

**Ingredient, % as is**	**Treatments**			
	**M**	**MS**	**MHT**	**MSHT**
Maize	56.6	28.4	56.6	28.7
Sorghum	–	26.7	–	26.2
Hydrolysable tannins	–	–	0.10	0.10
Wheat bran	10.0	14.0	10.0	14.0
Poultry byproducts meal	10.5	13.5	10.3	12.8
Bovine meat and bone meal	8.60	7.60	8.70	7.50
Poultry fat	5.20	4.60	5.20	4.70
Corn gluten 60% CP	5.00	1.10	5.00	1.90
Flaxseed	1.00	0.90	1.00	0.90
Digest^a^	1.50	1.50	1.50	1.50
Cellulose	0.90	1.00	0.90	1.00
Premix mineral/vitamin^b^	0.40	0.40	0.40	0.40
Salt	0.40	0.40	0.40	0.40
Potassium chloride	0.03	–	0.03	–
**Analyzed chemical composition, %DM basis**				
Dry matter	92.3	91.6	91.0	92.3
Starch	39.9	41.5	41.0	39.4
Crude protein	17.0	16.5	17.6	18.5
Acid hydrolyzed fat	9.25	8.54	9.33	8.09
Ash^c^	5.78	5.97	5.79	5.87
Crude fiber^c^	3.40	3.40	3.40	3.40
Total dietary fiber	21.4	21.3	20.7	23.1
Gross energy, kcal/kg	4,804	4,812	4,776	4,842
Gelatinization index of starch, %	87.9	93.1	97.2	84.9

*M, maize; MS, maize + sorghum; MHT, maize + hydrolysable tannins; MSHT, maize + sorghum + hydrolysable tannins*.

a *DTECH 8L, S.P.F. Argentina S.A., Argentina*.

b *Premix (supplied per kilogram of diet): vitamin A (10,800 UI), vitamin D3 (980 UI), vitamin E (60 mg), vitamin K3 (4.8 mg), vitamin B1 (8.1 mg), vitamin B2 (6.0 mg), vitamin B6 (6.0 mg), 12 vitamin (30 mcg), pantothenic acid (12 mg), niacin (60 mg), folic acid (0.8 mg), biotin (0.084 mg), manganese (7.5 mg), zinc (100 mg), iron (35 mg), copper (7.0 mg), cobalt (10 mg), iodine (1.5 mg), selenium (0.36 mg), choline (2.400 mg), taurine (100 mg), and antioxidant BHT (150 mg)*.

c *Calculated values*.

### Experiment 1: Digestibility Assay

#### Experimental Design

The assay was conducted as a balanced incomplete Latin square design as a model proposed by Ai et al. (18). Eight dogs were assigned in four treatments and three 10-day periods, with two dogs per treatment in each period, for a total of six replications per treatment, according to the recommendations of the American Association of Feed Control Officials protocol (19). The model for the balanced incomplete Latin square design (8 × 3) was:

$$\begin{array}{l} \text{yij}\left(\text{k}\right)=\text{μ }+\text{ Timej }+\text{ Dogj }+\text{ τk }+\;\varepsilon \text{ij}\left(\text{k}\right) \end{array}$$

in which yij(k) is observation ijk, μ is the overall mean, Time is the effect of row, Dog is the effect of column j, τk is the fixed effect of treatment k, and εij(k) is the random error with mean 0 and variance σ2. Gender (female and male) was used as a criterion for blocking, and body weight was used to randomize them in the treatments. Each period lasted 10 days, with 5 days for adaptation to the cage and experimental diet, followed by 5 days of total feces and urine collection and measurement of fecal and urinary pH. Between each period, 15 days of rest were provided to the dogs so they could exercise. In the rest period, dogs were fed M diet.

#### Sample Procedure

To establish the beginning and the end of each period of feces and urine collection, a gelatin capsule containing 1 g of iron oxide (III) Fe_2_O_3_ was orally given to the dogs. Feces were collected for 5 days, every 3 h except night time (12 h), and scored as follows: 1 = very hard and dry stool; 2 = hard, dry, firm stool; 3 = soft, moist stool, well formed; 4 = soft and shapeless stool; and 5 = liquid stool, diarrhea. The fecal score analysis was conducted by a single trained person using the WALTHAM Feces Scoring System (20). After daily collection, feces were weighed and stored in a freezer at −20°C until the end of the trial to perform analysis. Total urine collection was performed daily in the morning and then stored in plastic bottles containing 1 g of thimol (Synth, Diadema, Brazil), and the pH was measured. The urine total volume was measured and kept in a freezer at −20°C until analysis. The fecal pH was measured immediately after collection using 2 g of fresh feces diluted in 20 mL of distilled water using a portable pH meter (Digimed DM-22, Campo Grande, Brazil).

#### Chemical Analysis

Stool from each dog was thawed, homogenized, and dried in forced-air oven at 55°C for 72 h, according to the recommendations of the Association of Official Analytical Chemists (21). Feces, sorghum, and diets were ground through a 1-mm screen in a Wiley hammer mill (DeLeo Equipamentos Laboratoriais, Porto Alegre, Brazil) and analyzed for dry matter (DM—AOAC 934.01), acid hydrolyzed fat (AHF—AOAC 954.02; model 170/3, Fanem, Saõ Paulo, Brazil), crude protein (CP—AOAC 954.01; model TE 036/2, Tecnal, Piracicaba, Brazil), crude fiber (CF—AOAC 962.10; model MA 450/8, Marconi, Piracicaba, Brazil), and ash (21). Diets and sorghum were analyzed for total dietary fiber, according to Prosky et al. (22), and starch, according to Karkalas (23). The model for the gelatinization index of starch was:

$$\begin{array}{l} Gelatinization\;index\;\left(\%\right)\\ \;=\;\frac{\left(total\;starch-resistant\;starch\right)}{\left(total\;starch\right)}\;\times \;100 \end{array}$$

Urine samples were thawed and homogenized, and 150-mL aliquots were lyophilized (Micromodulyi-Fis; Thermo Fisher Scientifics Inc., Maryland, USA) for analysis of DM and gross energy (GE). Another 50-mL aliquot was collected for analysis of CP. Dietary, fecal, and urinary GE were determined using isoperibolic bomb calorimetry (calorimeter model C2000 basic, Ika-werke, Staufen, Germany). All analyses were performed in duplicate, assuming a coefficient of variation <1% for energy and <5% for the other analyzes. Ingredients, diets (Table 3), and feces were analyzed for phenolic compounds by gravimetric tests; total phenols and tannins were measured according to Makkar et al. (24) and CT based on Porter et al. (25). Based on the content of total tannins, condensed tannins, and total phenols in maize, sorghum, and wheat bran, an estimated value was calculated for each of the experimental diets (Table 3) in order to compare with the results obtained with the gravimetric tests.

**Table 3:** Phenolic compounds present in ingredients and experimental diets.

**Item**	**Total tannins^a^**	**Condensed tannins^b^**	**Total phenols^a^**
**Ingredients (raw material)**			
Maize	2.98	0.01	5.73
Sorghum	18.4	30.7	27.5
Wheat bran	2.96	ND	5.69
**Diets (after extrusion)**			
M	2.38 (1.87)	0.10 (0.01)	4.43 (3.60)
MS	2.50 (5.85)	0.40 (7.76)	4.34 (9.27)
MHT	2.50 (1.90)	0.09 (0.01)	4.57 (3.66)
MSHT	2.94 (5.73)	0.78 (7.56)	5.12 (9.08)

*ND, not detected; M, maize; MS, maize + sorghum; MHT, maize + hydrolysable tannins; MSHT, maize + sorghum + hydrolysable tannins*.

a *Values are expressed as g of tannic acid^−1^ kg DM*.

b *Values are expressed as g of leucocyanidin kg^−1^ DM*.

*Calculated values in parentheses based on the content of each ingredient, in % DM basis*.

#### Statistical Analyses

Data were analyzed using the ANOVA procedure of SAS 9.4 (SAS Inst. Inc., Cary, NC). Means were compared using Tukey's test at 5% probability (*P* < 0.05). The *P* < 0.10 was considered a tendency.

### Experiment 2: Postprandial Glycemia Assay

The dogs and the dietary treatments were the same as previously described for the digestibility assay.

#### Experimental Design

The dogs were adapted to the experimental diets for 11 days, then were fasted for 12 h inside the metabolic cages before starting the first blood collection. Immediately before starting the experiment, the cephalic vein was cannulated with a catheter BD ANGIOCATH 22″ (Becton, Dickinson and Company do Brasil, Curitiba, Brazil). Then, 1 mL of blood was collected in a tube containing 0.05 mL of sodium fluoride (LABTEST, Lagoa Santa, Brazil); this sample was used to determine the baseline glycemia at time 0. Dogs were fed, and dietary consumption was performed within 5 min. Sequential collections were started over 8 h after total consumption, at 5, 10, 15, 30, 45, 60, 90, 120, 180, 240, 300, 360, 420, and 480 min after food consumption. After each collection, the catheter was washed out with heparinized solution, and before each new collection, about 0.3 mL of blood was discarded.

#### Chemical Analyses

The tubes were centrifuged at 3,000 *g* for 10 min, and plasma was transferred to 1.5-mL Eppendorf tubes, cooled between 2 and 4°C, and analyzed in sequence. Blood glucose was analyzed by the enzymatic colorimetric method according to the manufacturer's instructions (Wiener Lab Group, Rosário, Argentina). All samples were analyzed in duplicate.

#### Statistical Analyses

The results were analyzed using repeated measures of variance on SAS 9.4 (SAS Inst. Inc., Cary, NC). The area under the curve (AUC), basal, maximum, average, and minimum glycemia, and maximum glycemic increase was calculated, and the means of each treatment were compared by Tukey's test (*P* < 0.05).

## Results

All experimental diets were well-consumed by the dogs without refusals.

There was a reduction in the phenolic compounds content, mainly in the diets with sorghum, MS and MSHT, after the extrusion (Table 3).

Dogs fed the diet containing both types of tannins together, CT and HT, consumed more phenol and tannins (*P* < 0.05) (Table 4). Phenolic compounds have not been found in feces of dogs fed the diets with HT, MHT, and MSHT, thus leading to the 100% apparent digestibility coefficients shown in Table 4.

**Table 4:** Phenolic compounds intake and digestibility of dogs fed the experimental diets.

**Item**	**Treatments**				***P*-value**	**SEM**
	**M**	**MS**	**MHT**	**MSHT**		
**Daily phenolic intake, g/day**						
Total phenols^1^	9.69^b^	8.97^b^	9.79^ab^	10.9^a^	0.0124	0.74
Total tannins^1^	5.21^b^	5.17^b^	5.35^b^	6.28^a^	0.0029	0.41
Condensed tannins^2^	0.22^c^	0.83^b^	0.19^c^	1.67^a^	<0.0001	0.08
**Apparent total tract digestibility, %**						
Total phenols^1^	95.2^b^	96.4^b^	100^a^	100^a^	<0.0001	0.30
Total tannins^1^	94.9^b^	95.3^b^	100^a^	100^a^	<0.0001	0.29
Condensed tannins^2^	97.3^c^	98.2^b^	100^a^	100^a^	<0.0001	0.28

*M, maize; MS, maize + sorghum; MHT, maize + hydrolysable tannins; MSHT, maize + sorghum + hydrolysable tannins; SEM, standard error of the mean*.

1 *Values are expressed as g of tannic acid^−1^ kg DM*.

2 *Values are expressed as g of leucocyanidin kg^−1^ DM*.

a,b,c *Means in the same row with different lowercase letters are significantly different (P < 0.05)*.

Dogs fed the experimental diets containing sorghum consumed less fat daily (*P* = 0.0009) (Table 5). The addition of sorghum containing CT or HT to the dietary treatments did not promote any differences in the coefficients of digestibility of nutrients and energy (*P* > 0.05).

**Table 5:** Nutrient intake and digestibility of nutrients and energy of dogs fed the experimental diets.

**Item**	**Treatments**				***P* value**	**SEM**
	**M**	**MS**	**MHT**	**MSHT**		
**Daily nutrient intake, g/day**						
DM	219	207	214	213	0.6606	15.5
OM	206	194	202	201	0.6344	14.6
AHF	20.2^a^	17.7^b^	20.0^a^	17.3^b^	0.0009	1.35
CP	37.2	34.1	37.7	39.4	0.0853	2.75
CF	7.44	7.03	7.28	7.26	0.6578	0.53
NFE	149	143	144	144	0.7376	10.5
Ash	12.6	12.3	12.4	12.5	0.9387	0.90
GE, kcal/day	1,051	995	1,023	1,034	0.7761	74.7
ME, kcal/day	164	158	164	160	0.5495	12.0
**Apparent total tract digestibility, %**						
DM	78.7	80.6	81.4	78.2	0.1363	2.81
OM	82.0	83.4	83.4	81.7	0.3642	2.02
AHF	85.8	86.2	88.3	83.2	0.0766	3.00
CP	78.1	78.4	82.0	79.5	0.1612	3.15
NFE	82.5	84.2	84.7	82.1	0.1457	2.41
GE	81.6	83.0	83.9	81.3	0.4170	2.05
**Nutritional value, kcal/kg**						
ME	3,755	3,831	3,834	3,755	0.3366	110

*M, maize; MS, maize + sorghum; MHT, maize + hydrolysable tannins; MSHT, maize + sorghum + hydrolysable tannins; SEM, standard error of the mean; DM, dry matter; OM, organic matter; AHF, acid hydrolyzed fat; CP, crude protein; CF, crude fiber; NFE, nitrogen-free extractive; GE, gross energy; ME, metabolizable energy*.

a,b *Means in the same row with different lowercase letters are significantly different (P < 0.05)*.

No differences were observed in the fecal and urinary characteristics analyzed (*P* > 0.05), except a tendency of diets with sorghum to increase fecal pH (*P* = 0.0948) (Table 6). Feces remained with adequate characteristics such as hard, dry, and firm stool. Dogs fed diets containing sorghum and HT had darkened feces and urine.

**Table 6:** Fecal and urinary characteristics of dogs fed the experimental diets.

**Item**	**Treatments**				***P*-value**	**SEM**
	**M**	**MS**	**MHT**	**MSHT**		
**Fecal characteristics**						
DM, %	33.5	34.3	35.8	35.6	0.3263	2.71
Output, g/day	141	118	110	131	0.2043	21.8
Output, g/day (DM)	46.7	40.2	39.5	46.7	0.2794	7.46
Fecal score^1^	2.23	2.12	2.02	2.01	0.3064	0.24
pH	6.34^b^	6.44^a^	6.34^b^	6.54^a^	0.0948	0.17
**Urinary characteristics**						
Volume, mL/day^2^	261	293	254	293	0.7620	99.9
pH	7.24	7.36	7.18	7.21	0.4611	0.29

*M, maize; MS, maize + sorghum; MHT, maize + hydrolysable tannins; MSHT, maize + sorghum + hydrolysable tannins; SEM, standard error of the mean; DM, dry matter*.

1 *Scored as: 1 = very hard and dry stool, 2 = hard, dry, firm stool, 3 = soft, moist stool, well formed, 4 = soft and shapeless stool, 5 = liquid stool, diarrhea*.

2 *Mean volume produced in 5 days*.

a,b *Means in the same row with different lowercase letters are significantly different (P < 0.10)*.

**Figure 1:**
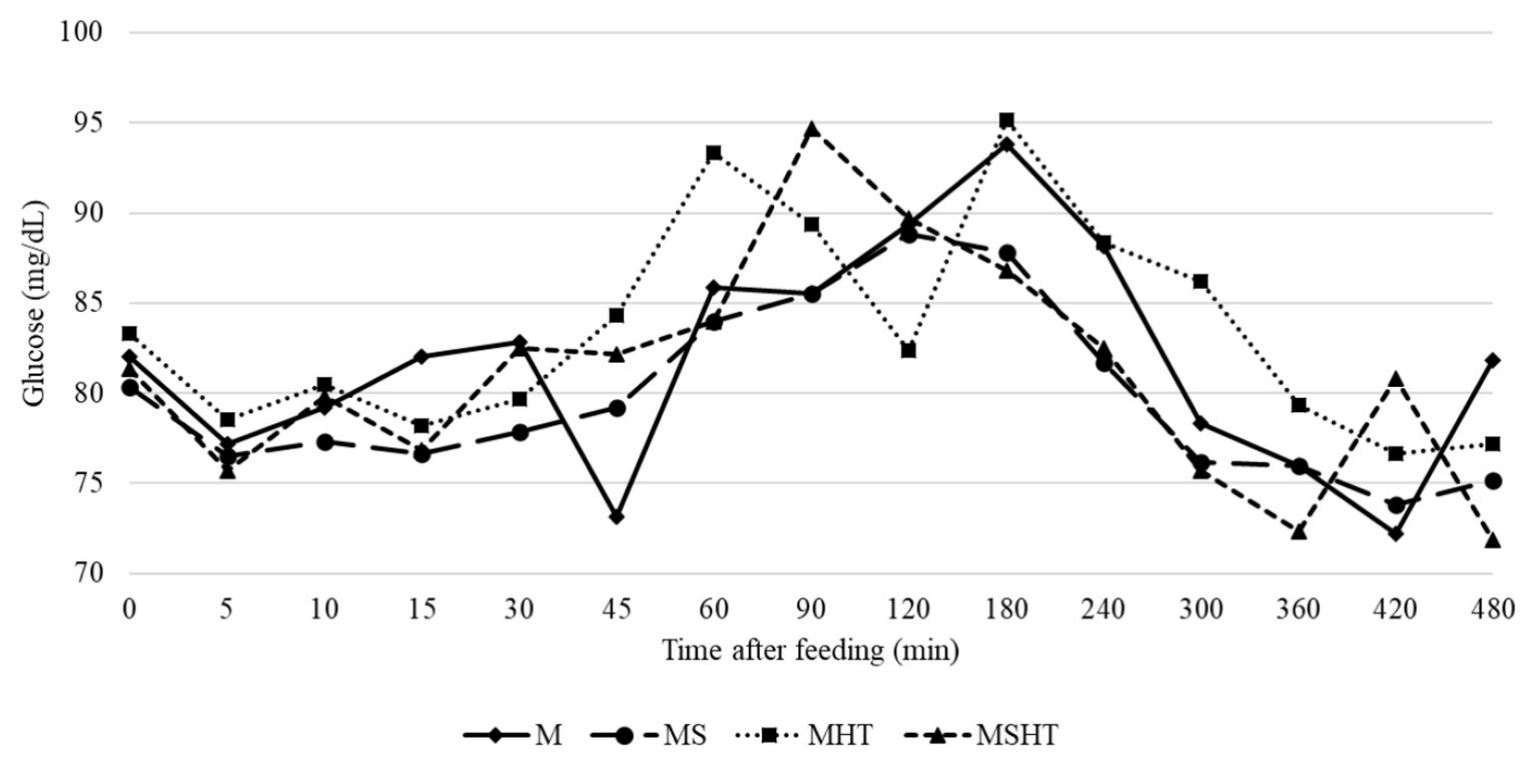
Average postprandial glycemic curves of dogs fed the experimental diets (mg/dl). M, maize; MS, maize + sorghum; MHT, maize + hydrolysable tannins; MSHT, maize + sorghum + hydrolysable tannins.

The glycemic items evaluated, e.g., area under the curve, basal, maximum, average, and minimum glycemia, and maximum glycemic increase, did not differ between the dietary treatments (*P* > 0.05) (Table 7). The average postprandial glycemic curves for the dietary treatments are illustrated in Figure 1.

**Table 7:** Area under the curve without basal glycemic area (AUC), plasma basal glucose concentration, plasma glucose concentration, and values of the glycemic peak of dogs fed the experimental diets.

**Item**	**Treatments**				***P*-value**	**SEM**
	**M**	**MS**	**MHT**	**MSHT**		
AUC total (0–480) mg/dl min	39,628	38,633	40,585	38,884	0.7211	3,168
Basal glycemia, mg/dl	82.0	80.3	83.3	81.3	0.9621	10.0
Maximum glycemia, mg/dl	95.5	97.0	101	96.0	0.8122	11.5
Average glycemia, mg/dl	82.7	80.2	83.8	82.0	0.8606	7.51
Minimum glycemia, mg/dl	70.0	66.7	69.7	69.5	0.5915	4.68
Maximum glycemic increase, mg/dl	13.5	16.7	18.0	14.7	0.8621	9.44

*M, maize; MS, maize + sorghum; MHT, maize + hydrolysable tannins; MSHT, maize + sorghum + hydrolysable tannins; SEM, standard error of the mean; AUC, area under the curve*.

## Discussion

Functional ingredients are highly pursued and evaluated in both human and animal nutrition. The main reason is that, besides nutritional value, these ingredients contain compound with metabolic effects, either promoting satiety or controlling the peak of glycemia after a meal, for example. The search for ingredients with these characteristics is valuable for pet food production, since obesity rates have increased over the years accompanied by associated diseases, such as diabetes mellitus. Based on previous evidence that sorghum containing tannins may interfere with digestive enzyme activity (26), we aimed to investigate the effects of condensed tannins, naturally present in sorghum, added to hydrolysable tannins on digestibility and whether these phenolic compounds could modulate postprandial glycemia, as it can affect absorption.

The inclusion of tannins may affect the palatability. The intake of tannins is related to a sensation known as astringency, caused by the interaction between salivary proline-rich protein, mucosal epithelium of the oral cavity, and tannins and characterized by the formation of complexes and precipitates, decreasing the saliva's lubricity and resulting in dryness of the mouth (9). However, Mole et al. (27) noted that dogs and cats produced small amounts of salivary proline-rich proteins, and they did not have tannin affinity *in vitro*. This may explain the good acceptance and voluntary intake of the experimental diets formulated to contain condensed and hydrolysable tannins (MS, MHT, and MSHT).

Phenolic compounds intake and metabolism in this study indicate that these molecules disappeared after extrusion, since after the thermal process, only a small content of total tannins and phenols and condensed tannins was detected by the method used. This fact was highlighted after a comparison between the estimated values for the diets and the values obtained by the analytical method, in which the negative impact of extrusion on tannins and phenols detection was found. The amount of phenolic compounds in all diets was similar after extrusion despite the presence of sorghum and HT. In fact, phenolic compounds can be thermally sensitive and are highly disposed to degradation during the heating process (28). Phenolic compounds exist both in free, extractable by solvent solutions, and bound, covalently bound with cell wall components and non-soluble in organic solvents, form mainly in carbohydrates requiring acid or alkaline hydrolysis prior to the extraction. Since approximately 80% of the phenolic compound present in sorghum is the bound form, this cereal requires treatment to increase accessibility and perform its bioactive functions (29). According to Masisi et al. (30), the biological potential of phenolic compounds depends on their bioaccessibility, absorption in the gastrointestinal tract, and their bioavailability *in vivo*. Therefore, it is indispensable to select the most appropriate heat treatment that minimizes potential losses in order to guarantee the bioactive responses.

The phenolic compounds content can be affected negatively by heat treatment according to the genotype of sorghum, mainly due to differences in flavonoid composition, and in general, extrusion is more deleterious than other types of cooking (31). On the other hand, extrusion decreased total proanthocyanidins and their oligomers and polymers, possible due to the complexation of proanthocyanidins with other molecules, and increased the amount of proanthocyanidins monomers and dimers, suggesting that this heat treatment helps the cleavage of high molecular weight compounds into lower molecular weight (31, 32). The increasing in monomer and dimer proanthocyanidins is important since they have greater bioavailability and effectiveness on oxidative stress (33). However, sorghum functional effects as antioxidant are attributed to the oligomers (34).

In this study, we selected sorghum containing CT and replaced maize with sorghum in order to improve the amount of CT in the diets. In addition, we used an HT obtained in the form of purified powder to easily incorporate in the formulation. However, due to its structure as esters, HTs are rapidly hydrolyzed in gallic acid or hexahydroxydiphenic acid and the parent polyol (35). In opposite, although CT can be degraded to anthocyanidins, under mild or anaerobic conditions, it remains stable (25). Based on our results, it is possible that extrusion modified total proanthocyanidins and their oligomers and polymers into other conjugated forms that were not possible to detect. Additionally, the lack of standardized and commercial methods makes the identification and quantification of HT and its metabolites difficult. Specific analytical methods are needed in order to allow the correct quantification to evaluate its effects *in vivo* (9). However, processing must be an important part of the disappearance of CT. The consumption of CT in the MS diet was ~50% of the consumption in diet MSHT, which was not expected. Adding these results to those of starch digestibility, it is possible to observe that MSHT presents a starch gelatinization index of 10% lower than those found in the MS diet. Considering that the diets were milled in the same place, the cooking processing must be the probable source of this variation. Therefore, heat and pressure can have some impact on the CT.

In the current study, no negative effects on digestibility were detected by the addition of sorghum containing CT or with the supplementation of HT. Similar results were obtained by Carciofi et al. (11), who did not find differences in nutrient intake and digestibility between dogs fed diets containing maize or sorghum. In addition, Kore et al. (36) did not observe differences in intake and digestibility of nutrients in dogs fed diets containing maize or sorghum when evaluating alternative cereals to rice. However, Twomey et al. (14) demonstrated that the inclusion of sorghum on dog diet reduced the digestibility coefficients of protein, dry matter, and gross energy compared to dogs fed maize-based diet. In addition, Murray et al. (13) did not found differences in ileal digestibility of nutrients between diets based on maize or sorghum, but the total tract digestibility of dry matter, organic matter, and crude protein were decreased in dogs fed the sorghum diet. This occurs probably because tannins, especially proanthocyanidins, have a property of forming complexes and inhibiting activity of enzymes, such as pectinase, amylase, lipase, protease, and β-galactosidase, impairing absorption of proteins and carbohydrates (37).

The comparison of alternative cereals, such as sorghum, with cereals containing a large amount of available starch leads to differences in the digestibility coefficients of nutrients and energy. This was evidenced in several studies comparing rice vs. sorghum and maize and may be attributed to structural differences in the starch–protein matrix of these cereals that, according to Hoseney (38), forms very strong bonds between the protein and starch components of maize and sorghum, which finally results in starch resistant to digestion (39). Teixeira et al. (15) observed impairing of apparent digestibility of dry matter, organic matter, crude protein, and fat, as well as on digestible and metabolizable energy of dogs fed diets with partial substitution of rice by sorghum.

In fact, the appropriate processing of cereals includes grinding to a particle size, known as mean geometric diameter (MGD), which is directly associated to starch gelatinization during extrusion. As reported by Bazolli et al. (12), smaller particle sizes allowed higher starch gelatinization and digestibility for diets based on maize and sorghum compared to coarsely ground maize and sorghum. Thus, correct grinding and extrusion at high temperatures allows to reduce the resistant starch present on maize and sorghum, improving its availability. All four experimental diets had starch gelatinization degree above 80%, which indicates adequate cooking during the extrusion.

Neither fecal or urinary characteristics were altered by the consumption of diets containing sorghum and HT. Due to its capacity to form complexes with enzymes, thus reducing the digestibility of nutrients, it was expected that the addition of sorghum promoted an increase in the fecal volume and a decrease in the DM of feces. In line with our findings, several studies have also found no differences between dogs fed diets containing maize and sorghum regarding fecal characteristics, such as fecal score, fecal pH, and fecal DM (11, 13, 14), with fecal score remaining as hard, dry, and formed stool as how we classified the stools in the present study. Down et al. (40) demonstrated that wild rodents consuming diets containing CT produced a more alkaline urine. This evidence probably could be found if we completely replaced maize with sorghum. There was a tendency to observe higher pH in dogs fed diets containing sorghum. The only aspect that differed in dogs fed diets containing CT or HT from the dogs fed the M diet was the darker coloration of feces and urine produced by dogs fed diets MS, MHT, and MSHT. It indicates that these phenolic compounds are absorbed by dogs, and their components are excreted in both feces and urine.

Finally, the glycemic parameters evaluated in this study did not change as we expected. Based on the results obtained by Carciofi et al. (11), in which dogs fed diets containing sorghum presented higher later meal response, characterized by a long and flat glycemic area under the curve, we hypothesized that dogs consuming diets with sorghum containing CT and HT would present a different response over the postprandial glycemia. The variety of sorghum selected to formulate our diets was considered as a high tannin grain (4.8% of polyphenol tannins). The contrasting results may be explained by the differences in the diets' chemical composition, including type of starch and granule structure and the fiber content. On the study conducted by Carciofi et al. (11), they replaced completely the source of starch by sorghum, and the levels of fiber, especially total dietary fiber (TDF), were not maintained similar between the diets, in which the maize-based diet had 9.4% of TDF while the sorghum diet had 14.1% of TDF. In the current study, the experimental diets were formulated to be isonutritive, so the levels of TDF were kept closer (M = 21.4%, MS = 21.3%, MHT = 20.7%, and MSHT = 23.1% of TDF). The total fiber could play a greater role in the digestion and glycemia compared to the tannins.

According to Clifford and Scalbert (41), compared to CT, the HT ones interact with proteins and form less stable bonds that can be metabolized by the intestinal microbiota, making them more soluble. Additionally, HT are absorbed mainly in the small intestine and less fermented in the colon (42). Thus, supplementation with HT in the current study may have been completely metabolized and, thus, not enough to promote some modulation in the glycemic response.

Based on the results obtained in this study and previously, many factors are associated with the modulation of the postprandial glycemic response in dogs. The use of sorghum, containing CT, and the purified HT extract did not negatively impact the digestibility of nutrients and energy and the fecal and urinary characteristics evaluated as previously reported in some studies. In fact, extrusion appears to have some impact on phenolic compounds that are not recovered by the same analytical method. Thus, the phenolic compounds could not be related to any interference in the evaluated responses since they were not detected in the same amount supplied in the diets before extrusion. The partial substitution of maize for sorghum and the addition of HT do not interfere in the postprandial glycemia response in adult dogs. Maize and sorghum are capable of providing similar amounts of nutrients to dogs. The substitution of 50% of maize for sorghum can be a practice applicable in diets without losing the nutritional value of the final diet.

## Data Availability Statement

The raw data supporting the conclusions of this article will be made available by the authors, without undue reservation.

## Ethics Statement

The animal study was reviewed and approved by The Institutional Animal Care and Use Committee at the Universidade Federal do Rio Grande do Sul, protocol number 26275.

## Author Contributions

LTe, LTr, and AK designed the study. LTr acquired the grant and provided logistical support and graduate student mentorship. LTe, LTr, and GM conducted the animal trial and sample collection. CP conducted statistical analysis. CP, LTe, and LTr wrote the manuscript. All authors have read and approved the manuscript.

## Conflict of Interest

The authors declare that the research was conducted in the absence of any commercial or financial relationships that could be construed as a potential conflict of interest.
